# Flame-forged divergence? Ancient human fires and the evolution of diurnal and nocturnal lineages in moorish geckos

**DOI:** 10.1016/j.isci.2024.111715

**Published:** 2024-12-30

**Authors:** Domenico Fulgione, Danilo Russo, Eleonora Rivieccio, Valeria Maselli, Bice Avallone, Alessandro Mondanaro, Giorgio Giurato, Maria Buglione

**Affiliations:** 1 Department of Biology, University of Naples Federico II, 80126 Naples, Italy; 2 Animal Ecology and Evolution Laboratory (AnEcoEvo), Department of Agricultural Sciences, University of Naples Federico II, 80055 Portici, Naples, Italy; 3 Department of Humanities Studies, University of Naples Federico II, 80138 Naples, Italy; 4 Department of Earth Sciences, University of Florence, 50121 Florence, Italy; 5 Department of Medicine, Surgery and Dentistry Medicine, University of Salerno, 84081 Salerno, Italy

**Keywords:** Zoology, Phylogenetics, Evolutionary biology

## Abstract

Using a multidisciplinary approach, we investigated whether human-controlled fire has historically influenced temporal niche partitioning between dark-diurnal and pale-nocturnal lineages of the Moorish gecko (*Tarentola mauritanica*). The pale-nocturnal variant exhibited lower skin melanin levels, smaller and fewer melanosomes, and lower plasma α-Melanocyte Stimulating Hormone levels than its dark-diurnal counterpart. Mitochondrial genome analyses indicated that the common ancestor of these gecko lineages diverged approximately 6,600 years ago, coinciding with the transition of modern humans from nomadic hunter-gatherers to settled agricultural societies. Species distribution models suggested coexistence between humans and geckos during the emergence of these lineages. Additionally, we demonstrated that fire attracts phototactic arthropods, concentrating prey resources. These findings imply that human-controlled fire may have created a novel foraging niche for pale-nocturnal geckos, likely driving the divergence of the two variants.

## Highlights

• *Tarentola mauritanica* shows two variants: a dark-diurnal and a pale-nocturnal form
• According to mtDNA, these forms began diverging ca. 6,600 years ago
• Human-controlled fire may have created a niche droving the split between these gecko forms
• This phenomenon may represent an incipient speciation driven by human civilization

## Graphical abstract

**Figure:**
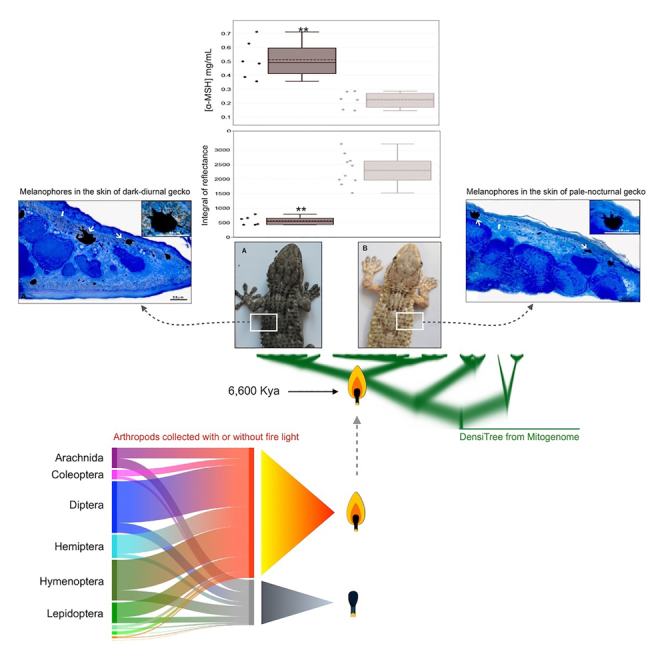

## Introduction

Humans are the only animal species capable of changing the environment globally.^1^ Human action has largely affected the fate of wildlife throughout humankind’s history, promoting profound changes in community composition and geographic ranges.^2^ Humans have also caused numerous extinction events,^1^^,^^3^ filtering out many species from human-altered landscapes where more opportunistic species persist or thrive,^4^^,^^5^ and not infrequently exerting selective pressures that have led to new phyletic lineages and speciation.^6^

Artificial lighting at night (hereafter, ALAN) is one of the most pervasive forms of environmental alteration caused by humans. Numerous organisms adjust their behavior and life cycles according to the availability of light across various time frames, ranging from daily to seasonal cycles. By introducing changes to the natural cycle of light and darkness, ALAN significantly impacts several crucial aspects of animal existence,^7^^,^^8^ especially affecting nocturnal species, whose sensitivity is evident as they either actively avoid light, as seen in most bat species,^9^^,^^10^ or are attracted to it, as in numerous nocturnal insects showing positive phototaxis (e.g.,^11^).

While there is much knowledge about the global, overwhelming impact of ALAN (e.g.,^12^) the effects of the first form of human-generated lighting, fire, are practically unknown. The intentional use of fire traces back to the Middle Pleistocene of the Levant^13^^,^^14^ but spread as a common practice soon afterward.^15^ The earliest use of fire may have been for cooking and repelling predators, but it also facilitated social interactions. The use of fire likely became widespread during the transition of modern humans to settled agricultural communities, associated with increased human density and a new lifestyle. This may have led to a “daylight extension,” in which humans have developed an activity peak during late evening hours, an unusual condition compared to the mammalian standard, including other primates.^16^^,^^17^

Besides changing the microclimate, fire generates light at night, when numerous phototactic insects may get lured and fall victim to opportunistic predators exploiting the new environmental conditions and their associated food bonanza. However, no study has explored the possibility that fire has historically created a previously untapped foraging niche suitable for insectivorous vertebrates. Although there is no way of retrospectively investigating the soundness of this hypothesis, several clues may be gathered at least to test the potential influence of ancient human-governed fires on animal ecology and evolution.

We selected the Moorish gecko (*Tarentola mauritanica*) as a case study to reach this goal. This small saurian is widespread in North Africa and Europe and has the widest range of all gecko species in the Mediterranean Basin.^18^ Because of the close relationship with humans, the species has been accidentally introduced to the Balearic Islands (Spain), Madeira (Portugal), some Balkan islands, Crete, and South America.^19^^,^^20^ European populations of Moorish geckos diverged from the central Morocco clade during the early Pliocene, around 4.14 Mya. The colonization of the Iberian Peninsula by Moorish geckos from North Africa could have occurred during the Messinian Salinity Crisis or after the opening of the Strait of Gibraltar during the early Pliocene.^21^

**Figure 1:**
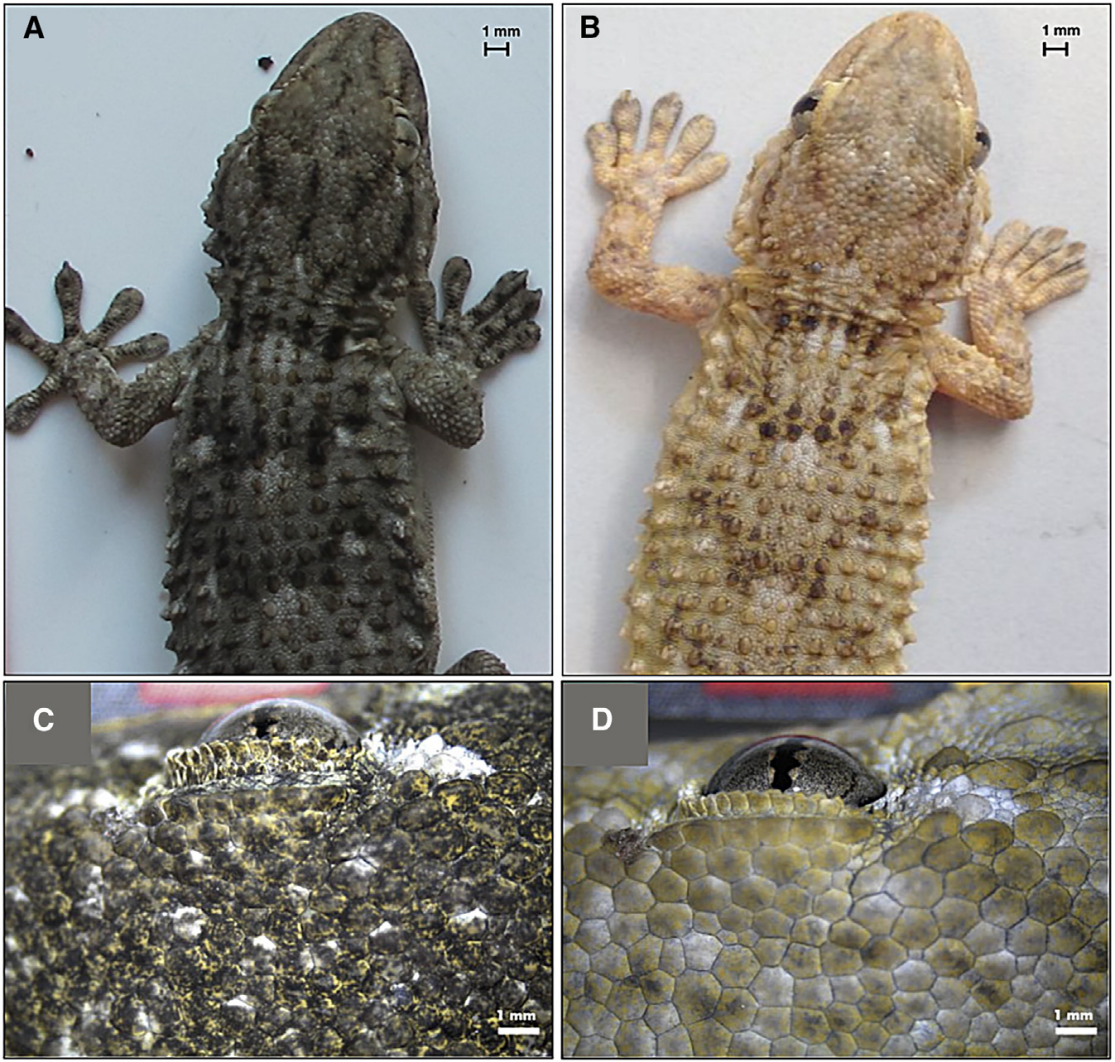
Dark-diurnal (A) and pale-nocturnal (B) Moorish geckos (*Tarentola mauritanica*) morphotypes The two lineages occupy distinct ecological niches: the diurnal form inhabits trees, while the nocturnal variant prefers building walls at night. Insets (C) and (D) show the plasticity of a dark-diurnal gecko changing skin color on different substrates (see^25^ for more details). The bar scale is representative of 1mm (see also Figure S1).

The Moorish gecko is active during diurnal and nocturnal hours, occupying several ecological niches, such as tree trunks, houses, and stone walls.^22^^,^^23^^,^^24^ This species represents an interesting example of phenotypically plastic variation, highlighted by its ability to adapt skin color according to the substrate.^25^ Two distinct and sympatric populations can be easily distinguished^22^ (Figure 1): the “dark diurnal gecko,” which mainly lives on trees, and the “pale nocturnal gecko,” found in human settlements on walls, especially near artificial lights, to increase the chances of prey capture.^22^^,^^26^^,^^27^ These two phenotypes are likely adapted to improve camouflage and are observed in many areas of the Mediterranean Basin (Figure S1). Experimental work showed that pale-nocturnal geckos are less exposed to predation on walls (their natural substrate) at night while dark-diurnal geckos are less attacked in trees (their natural substrate) during the day.^22^ The dark color of diurnal geckos may have primarily evolved to improve thermoregulation.^28^^,^^29^^,^^30^ We propose that as Neolithic humans spread across Europe, especially the Mediterranean, human-controlled fire played a significant ecological role, introducing a new foraging opportunity for Moorish geckos. This opportunity arose from positive phototactic arthropods being attracted to the light provided by fire. It is currently unknown whether nocturnal Moorish geckos are the ancestral form from which diurnal geckos have evolved, or vice versa. We argue that both evolutionary paths are conceivable and may be associated with the spread of human-generated fire. The following scenarios illustrate this association:

*“Out of the dark” scenario*: the ancestral form was nocturnal and largely exploited human-generated fire. However, Moorish geckos are highly territorial and aggressive,^31^^,^^32^ so only some dominant individuals controlled the food bonanza offered by the fire. Less competitive individuals were excluded from these profitable foraging sites and confined to peripheral sites whose food availability was impoverished by the “vacuum effect” exerted by the fire. The more phenotypically plastic geckos exploited their capacity to adapt their color to daytime and mitigate predation in daylight, so they conquered a novel diurnal niche, leaving the less plastic, dominant individuals associated with humans and their fires.

*“Into the dark” scenario*: the phenotypic variation in skin color shown by this species has set the scene for the evolution of a night-adapted lineage and led to the split between dark and pale geckos. The direct ancestors of the nocturnal form may have included particularly pale individuals whose coloration protected them from nocturnal predators^22^ while allowing them to exploit habitats around human-set fires in villages, near walls of the houses or rocky surfaces, as successful foraging sites. By attracting positively phototactic insect prey, illuminated surfaces may have offered a new foraging niche that the geckos have successfully exploited. This may have led to the evolution of a paler, nocturnal, and synanthropic lineage that has ever since accompanied our species and the creation of urban settlements up to modern times.

To explore these evolutionary scenarios, we formulated the following hypotheses and predictions:

(1) Although the two forms, diurnal and nocturnal respectively, retain the ability to vary the level of darkness/lightness to some extent,^22^ nocturnal geckos are characterized by a more marked depigmentation resulting from their high adaptation to the nocturnal niche. We predict that the two forms will differ in skin reflectance in response to the blood levels of α-MSH (α-Melanocyte Stimulating Hormone) and the size and numerosity of melanophores, overall revealing distinct structural features between the lineages adapted to the two temporal niches.
(2) During the Holocene, a wave of human populations from the Middle East spread in Europe via Anatolia.^33^^,^^34^^,^^35^ Studies based on radiocarbon suggest that this migration wave spread farming practices into the region, initiating the Neolithic revolution in Europe.^36^^,^^37^^,^^38^^,^^39^^,^^40^ The advent of farming practices brought about the widespread use of fire, potentially creating a unique foraging niche for a specialized gecko lineage. It is plausible that Moorish geckos and humans encountered each other in the Mediterranean region during this period, assuming they inhabited the same areas. Consequently, we predict that the common ancestor in the two gecko lineages occurred during sympatry with humans.
(3) Fire may significantly increase prey availability when lit near wall surfaces by attracting arthropods eaten by the nocturnal gecko form. Preliminary work has established a clear diet difference between diurnal and nocturnal geckos, with the former mostly preying on Formicidae, and the latter feeding on moths, small dipterans, and orthopterans (pers. obs.). We, therefore, predict that such potential prey will be more abundant near fire than under dark conditions, acting as the control.

## Results

### Skin reflectance, dermal melanin content, and α-MSH levels differ between pale-nocturnal and dark-diurnal geckos

**Figure 2:**
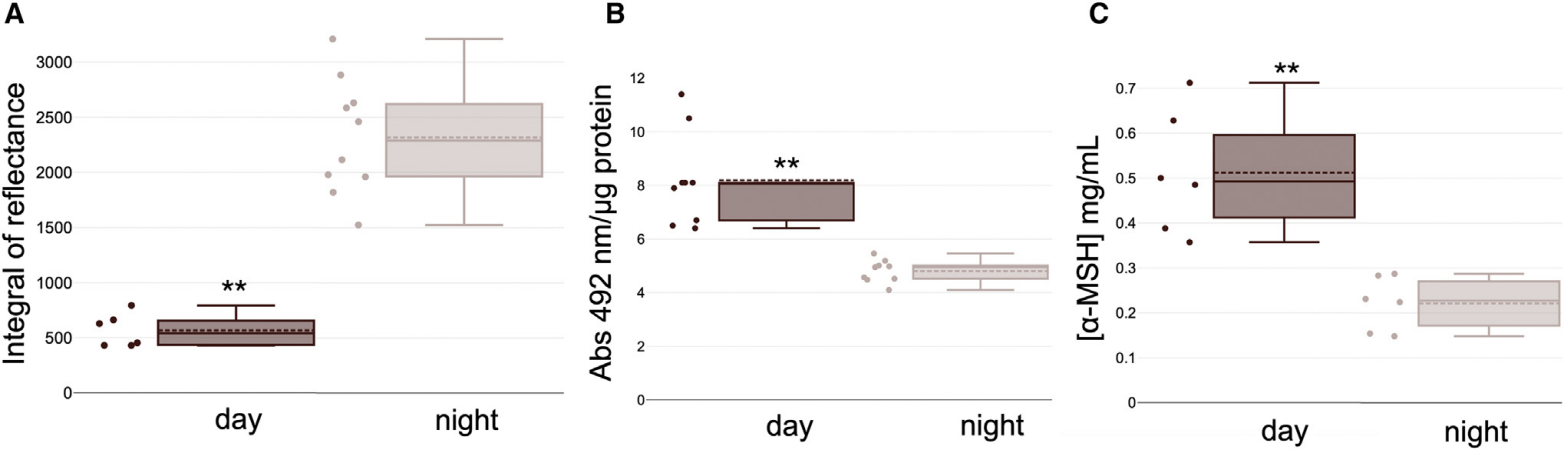
Comparisons between the two geckos’ morphotypes Comparisons between dark-diurnal (“day”) and pale-nocturnal (“night”) Moorish geckos (*Tarentola mauritanica*) for (A) Integral under skin reflectance curve, (B) spectrophotometric skin melanin assay (ng/μg protein) and C) α-MSH hormone blood concentration (mg/mL). Data are presented as boxplots showing the mean (dotted line), median (bold line), interquartile range (box), and whiskers representing ±1 standard deviation. Individual data points are shown as dots. One-way ANOVA tests, ∗∗*p* < 0.001 (see also Tables S1–S3).

Pale-nocturnal geckos showed a significantly lower skin reflectance than dark-diurnal geckos (df = 1, 14; *F* = 62.37 *p* = 0.00001) (Figure 2A; Table S1). Accordingly, both dermal melanin production (df = 1, 16; *F* = 32.3; *p* = 0.00003) (Figure 2B; Table S2) and α-MSH’s blood levels (df = 1, 10; *F* = 22.63 *p* = 0.0007) (Figure 2C; Table S3) differed between forms.

### Pale-nocturnal geckos show smaller and less abundant melanophores than dark-diurnal individuals

**Figure 3:**
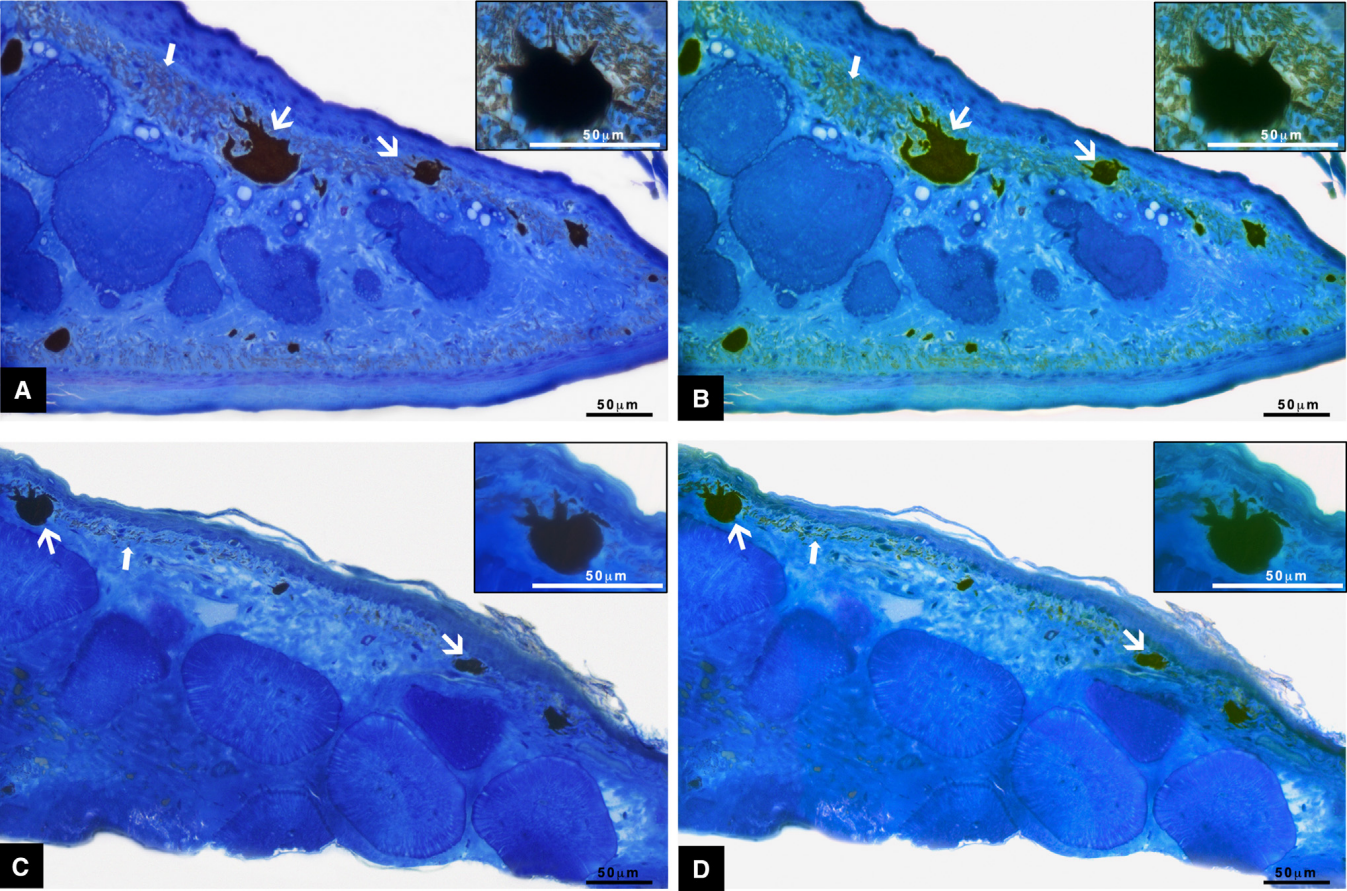
Skin sections stained with toluidine blue (A) Skin from the dorsal area of dark-diurnal Moorish geckos (*Tarentola mauritanica*), with single melanophores (inset). A high concentration of melanophores (thick arrow) is localized in the dermal region, along with a thick layer of melanin granules (arrow). (B) Digitally stained version of image A to emphasize melanophores and melanin pigment. (C) Skin from the dorsal area of a pale-nocturnal gecko, with a single melanophore (inset). (D) Digitally stained version of image C to emphasize melanophores and a layer of melanin pigment. The bar scale is representative of 50μm.

The comparison of histological skin samples taken from the dorsal surface of pale-nocturnal and dark-diurnal Moorish geckos revealed clear differences, in agreement with our hypothesis. Dark phenotype geckos exhibited numerous, predominantly large melanophores. Additionally, a thick and continuous layer of pigment granules was observed in the connective tissue of the underlying dermis. In contrast, the skin of pale-nocturnal specimens displayed remarkably reduced melanophores in size and abundance, and the layer of pigment surrounding them was sparse (Figure 3).

### Significant Spatial Overlap Between Humans and Geckos at the Time of Gecko Divergence

**Figure 4:**
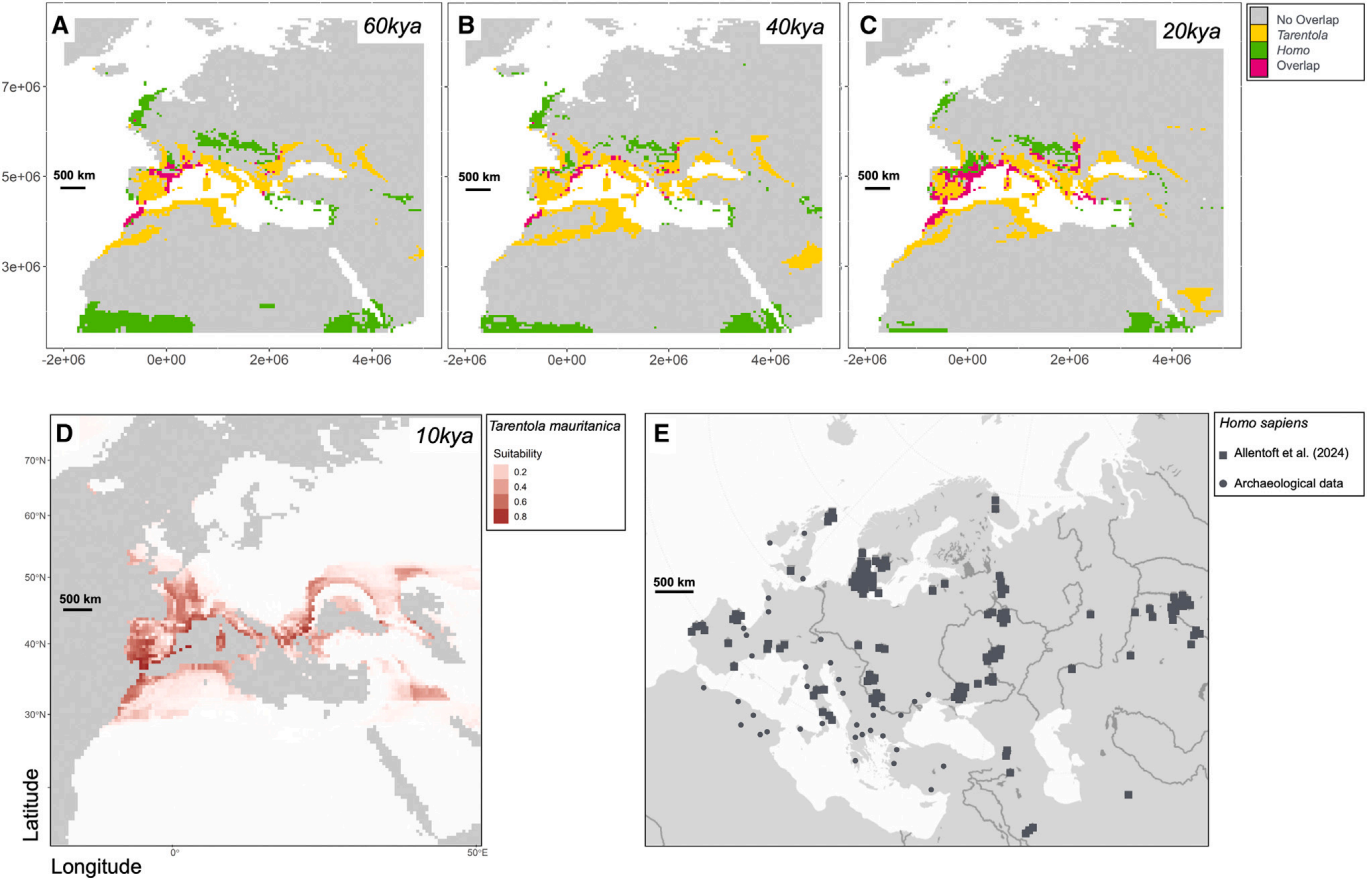
Past sympatry between *Tarentola mauritanica* and *Homo sapiens* (A, B, and C) Spatial distribution models of *T. mauritanica* and *H. sapiens* and their overlap during the time interval 0.06 Myr−0.02 Myr. The models indicate significant overlap in potential distribution within the Mediterranean region, supported by a linear mixed-effect model demonstrating a positive relationship between potential distributions of the two species. (D) Spatial distribution model of *T. mauritanica* approximately 10,000 years ago (Boyce Index, Spearman correlation test and the linear mixed-effects model *p* < 0.001). (E) Distribution of the 317 ancient genomes of *H. sapiens* mainly from the Mesolithic and Neolithic periods (filled squares, modified from^41^) and further archaeological records (see also Figure S2; Table S4). The bar scale is representative of 500 km of distance.

The SDMs developed for humans and geckos were projected into the past to estimate their potential distribution in the Mediterranean area during the 0.06 Myr−0.01 Myr interval covering the estimated divergence time between the dark and pale gecko forms. These models exhibited high predictive performance. Specifically, the SDM developed for humans showed an AUC of 0.818 (S.D. = 0.014) and a TSS of 0.502 (S.D. = 0.028). Similarly, the SDMs developed for *T. mauritanica* achieved high performance, with an AUC of 0.96 (S.D. = 0.002) and a TSS of 0.803 (S.D. = 0.008). We assessed the spatial correlation between humans and the Moorish gecko in the past using the Boyce Index, obtaining a Spearman correlation value of 0.422. Furthermore, the linear mixed-effects model revealed a positive and significant (angular coefficient of the distribution, a = 0.6; *p* < 0.001) relationship between *H. sapiens* and *T. mauritanica* potential distributions (Figure S2). The niche overlap between *T. mauritanica* and *H. sapiens* was highest in Southern Iberia and north-western Africa (Figure 4). Additionally, the model exhibited a relevant age-driven random effect structure, with increasing overlaps toward more recent time intervals (*p* = 0.016, Figure 4).

### Mitogenome analysis traces the origin of pale-nocturnal and dark-diurnal lineages approximately 6,600 years ago

**Figure 5:**
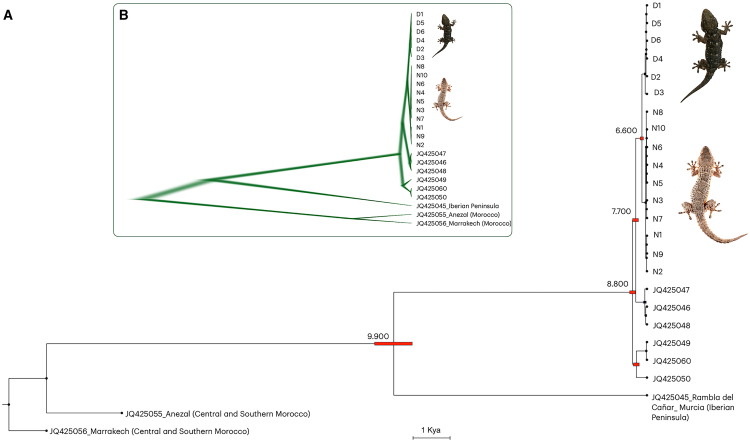
Phylogenetic characterization (A) Phylogeny and divergence time estimation derived from molecular-clock analysis of 10 pale-nocturnal (N1-N10) and 6 dark-diurnal (D1-D6) *Tarentola mauritanica,* in addition to other Italian haplotypes.^42^ Divergence times for the two gecko populations were calculated using the complete mitochondrial genome. The red bars on the nodes represent the 95% credibility intervals of the estimated posterior distributions of the divergence times. The bar scale is representative of 1 Kya, thousand years ago. (B) DensiTree. All trees created in the analysis (except the burn-in phase) are displayed. Trees with the most common topology are highlighted in dark green, and trees with the second most common topology in light green.

The phylogenetic analysis based on the complete mitochondrial genome revealed that the Moorish gecko’s pale and dark populations are monophyletic, sharing a common ancestor estimated to have emerged approximately 6,600 years ago (Figure 5A). Samples from Calabria, Apulia, and northern Campania were identified as sister taxa to all pale-nocturnal and dark-diurnal geckos included in the analysis.

The tree set with a dominant topology in the Bayesian analyses provided the distribution and origination time of the Most Recent Common Ancestor (tMRCA) (Figure 5B). All post-burn-in trees are shown with their estimated branch lengths and topologies, where the fuzziness of the horizontal plane of the branches visualizes variation in branch lengths across the trees. This reconstruction also demonstrates strong support for separating pale-nocturnal and dark-diurnal geckos, as indicated by the topology (dark green branches). In contrast, increased uncertainty in the tMRCA is represented by light green shading.

### Fire-illuminated sites provide higher prey availability

**Figure 6:**
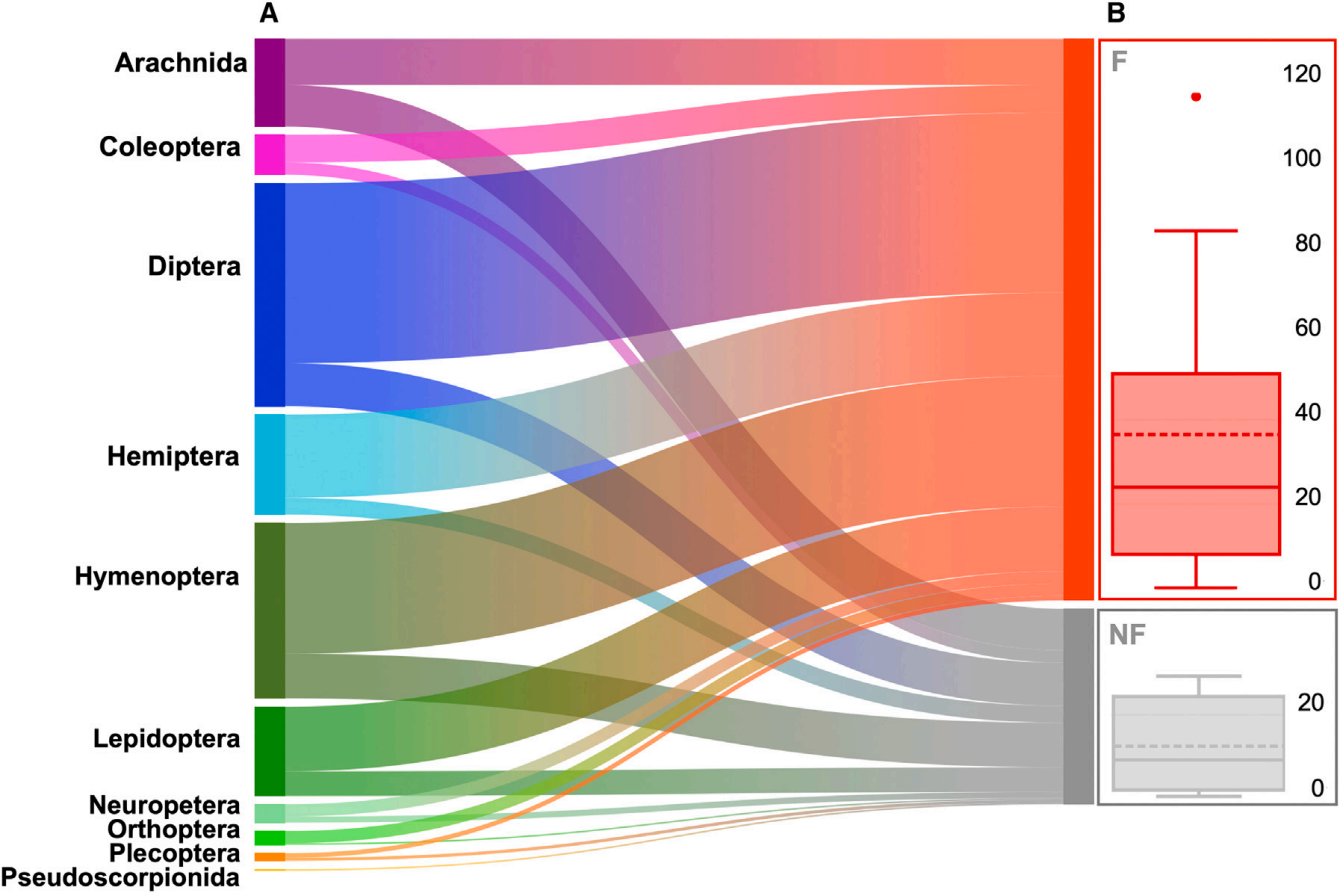
Characterization of trophic availability Numbers of arthropods collected from walls exposed to fire (F) vs*.* walls not exposed to fire (NF). (A) Sankey diagram connecting numbers of individuals collected by order with treatment. (B) The total number of individuals captured under the two conditions is shown. Data are represented as boxplots with the mean (dotted line), median (bold line), interquartile range (box), and whiskers representing ±1 standard deviation. (two-tailed t-test; t = 2.50; d.f. = 9; *p* < 0.05) (see also Table S5).

The arthropods sampled at the walls illuminated by fire differed significantly from those recorded at the unlit walls (Figure 6). Specifically, we recorded higher numbers of arthropods at fire-illuminated sites considering the total number of individuals at the order level (two-tailed t-test = 2.50, *d.f.* = 9, *p* = 0.033) (Table 1). Samples were dominated by moths, mosquitos, grasshoppers, wasps, ants, and true bugs (see also Table S5).

**Table 1:** Paired sample t-test results for comparison between arthropods collected at walls exposed to fire vs*.* unlit walls

Taxon	d.f.	t	*p*
Diptera	63	4.75	0.00001^a^
Orthoptera	63	2.42	0.01840^a^
Hymenoptera	63	3.31	0.00152^a^
Hemiptera	63	4.39	0.00004^a^
Lepidoptera	63	2.69	0.00894^a^
Coleoptera	63	1.74	0.08638
Neuroptera	63	1.27	0.20840
Arachnida	63	0.38	0.70040
Plecoptera	63	0.44	0.65827
Pseudoscorpionida	63	1.00	0.32113

a statistically significant difference (*p* < 0.05). d.f. = degrees of freedom.

## Discussion

The existence of Moorish geckos that are active during the night or daytime, and their extreme color variation, have long been recognized in the scientific literature (e.g.,^43^^,^^44^^,^^45^^,^^46^^,^^47^^,^^48^). However, no study has explored the phylogenetic history and the adaptations of the two forms. Although focusing on a defined geographical area, our study can be considered representative of all other populations that feature these two morphotypes (Figure S1).

### Did human-controlled fire influence the temporal niche partitioning in Moorish geckos?

We used a multidisciplinary approach to demonstrate that the two gecko variants have undergone phenotypic divergence over their evolutionary paths.

The pale-nocturnal form has smaller and fewer melanosomes and is associated with lower levels of α-MSH than the dark-diurnal form. Additionally, mitogenome analysis revealed that the pale-nocturnal lineage split from the dark-diurnal lineage around 6,600 years ago, making it unclear which of the two forms appeared first. However, molecular dating indicates that both forms diverged when Neolithic humans spread across Europe, coinciding with the use of fire in association with human settlement.

The area where we carried out our study is characterized by an abundance of caves long inhabited by humans (^49^^,^^50^). Charcoal analysis from the Cilento coast reveals a significant, human-driven shift in vegetation between 5600 and 7490 years ago (^51^ and G. Di Pasquale, pers. comm.) overlapping with the divergence time frame we estimated for the two gecko variants. The charcoal samples were obtained from stratified deposits inside caves, providing evidence of fire use within these sites. The presence of fire, charcoal remains and various archaeological materials, serves as clear evidence of human activity in these caves. Consequently, strong evidence suggests that humans extensively used caves and controlled fires in these underground sites, likely supporting our light-related evolutionary hypothesis.

This probably facilitated the evolution of the nocturnal lineage by enhancing the availability of suitable arthropod prey for pale geckos, thereby providing a new and previously unexploited foraging niche. Also, our spatial modeling exercise confirmed sympatry between humans and geckos when the two lineages arose. Finally, we showed that fire lit near walls mimicking a natural rock surface considerably increased the amount of arthropod food, attracting selectively insects such as moths, mosquitos, and wasps that are potential prey for the pale variant.

Overall, this multifaceted body of evidence suggests that ancient fires used by humans may have facilitated the separation between the two gecko variants leading to the situation we observe today. However, we could not fully clarify whether the two lineages originated from diurnal or nocturnal ancestors. While human-controlled fire may have represented an environmental novelty providing an important foraging niche at night, the evidence we gathered is still compatible with either scenario. The presence in Italy of geckos since the Upper Pleistocene is supported by fossil remains^43^ and excludes its historical, anthropogenic introduction in the country, in agreement with previous phylogenetic work.^44^

The timing of human control over fire remains a contentious issue.^45^^,^^46^ Evidence from Europe suggests that Neanderthals and early modern humans already used controlled and consistent fire starting from the Middle Pleistocene onwards in Europe, and from the Late Pleistocene in Europe and South Africa, for purposes beyond warmth and cooking.^47^^,^^48^ However, the hypothesis that controlled fires became prevalent upon the arrival of modern humans in Europe, particularly in the Mediterranean, is highly plausible.

We verified that the phenotypic divergence between the two gecko variants may have occurred broadly across the Mediterranean. However, we interpret our results cautiously, as the genetic samples used in this analysis are restricted to our study area. Expanding the sampling to include other Mediterranean regions (e.g., Spain, Portugal, France) would help clarify whether this phenomenon reflects a broader pattern or is a result of recent local adaptation. Such an expanded analysis should adopt the same multidisciplinary approach we used to investigate the two populations in our study area. Indeed, phylogenetic analyses incorporating sequences from outside our study area would either complicate or reinforce the hypothesis of a local phenomenon. Moreover, in a pan-Mediterranean analysis, it is important to consider that this species has been widely translocated by humans, often unintentionally.

### The “out of the dark” scenario

The hypothesis of a nocturnal ancestor that has led to a first pale-nocturnal lineage and later to a diurnal descendant is certainly parsimonious and agrees with the fact that based on a phylogenetic analysis of temporal activity patterns, nocturnality would be an ancient trait appearing at the base of the gecko tree.^52^

Geckos and skinks independently evolved nocturnality, diverging from the ancestral diurnality typical in lizards,^53^ with noticeable metabolic^54^^,^^55^ and sensory^56^ adaptations to the nocturnal niche. While the spread of human-controlled fire may have represented a strong advantage for more night-specialized phenotypes, these may have monopolized such preferred foraging sites excluding other less competitive individuals. In behavioral studies of *T. mauritanica* foraging near artificial lights, large individuals exclude smaller and younger geckos from key foraging sites,^57^ and a similar situation is conceivable near the fires set by ancient humans. On the other hand, living on the periphery of such sites for subordinate geckos constrained in their marginal territories may have been highly unprofitable because of the “vacuum effect” caused by the fire, attracting arthropod food and depleting the surrounding areas. This situation is well-known today in response to artificial lighting. It is regarded as one of the adverse effects of light pollution on natural environments, favoring a few opportunistic, light-tolerant predators at the expense of light-averse species whose dark habitat is continuously impoverished.^10^^,^^58^ The process has been proposed as the way light-tolerant common pipistrelles (*Pipistrellus pipistrellus*) have outcompeted light-averse lesser horseshoe bats (*Rhinolophus hipposideros*) in Switzerland, contributing to the decline of the latter species.^59^ Under such a scenario, the most phenotypically plastic individual geckos, capable of acquiring a sufficiently dark color to shift their temporal niche to the daytime, might have led to the evolution of the diurnal lineage observed today, escaping the strong competition by the nocturnal dominants.

### The “into the dark” scenario

The alternative “into the dark” scenario we considered would originate from a dark-diurnal ancestor^56^^,^^60^ from where the pale-nocturnal lineage, and later, the dark-diurnal lineage would evolve. During the evolutionary path leading to the current nocturnal specialists, an ancestral dark form would have lost the ability to conspicuously change its coloration, fixing a pale phenotype that would effectively mitigate predatory pressure at night.^22^ The attainment of the pale phenotype involved a reduction in α-MSH hormone levels and alterations in the number and size of melanosomes in the dermis. In other words, predators would have exerted strong selective pressure, killing all individuals whose coloration was not pale enough to fade their contour on rock walls at night, leaving only the palest ones on the scene. The latter individuals were probably those exhibiting a greater ability to lighten their color,^25^^,^^61^ and subsequently underwent depigmentation adapting to nocturnal life. The paleo-synanthropic relationship between geckos and humans has persisted into modern times. Today the pale-nocturnal gecko inhabits urban areas, often waiting for prey near artificial light sources. Although nocturnal lizards such as geckos may be active at body temperatures that are considerably lower than those characterizing activity in diurnal lizards,^62^^,^^63^ nocturnal species may still experience suboptimal locomotion in the cold of the night,^62^^,^^63^^,^^64^ which might, in theory, limit successful foraging. However, locomotion in nocturnal lizards is surprisingly efficient, as they outrun 3-fold diurnal lizards and show low-temperature performances like those of diurnal lizards at higher temperatures. This efficiency is attributed to a low minimum cost of locomotion^62^^,^^65^ and higher metabolic rates at low temperatures.^54^^,^^55^ In the case of pale geckos, hunting near fires might have represented a further way of warming up and achieving even higher locomotion performances.

The role of phenotypic plasticity in promoting the diversification of new lineages has been reconsidered in recent times,^66^ and today is seen as potentially important albeit its mechanisms are still debated (e.g.,.^67^^,^^68^^,^^69^^,^^70^^,^^71^^,^^72^ Phenotypic plasticity might serve as a protective mechanism against environmental fluctuations, fostering the persistence of populations.^66^^,^^73^^,^^74^ On the other hand, phenotypic plasticity could lay the groundwork for a process called “genetic assimilation,” where a phenotype becomes integrated into the genotype, potentially resulting in a loss of plasticity known as “canalization.”^75^ Under the “into the dark” scenario, the plasticity still observed in the dark-diurnal lineage and hypothetically shared with its diurnal ancestor facilitated the initial colonization and survival of the population in a new environment, allowing time for subsequent genetic adaptation to fine-tune responses to this environment. The plastic capabilities of the phenotype can thus respond to selection, anticipating the adaptation process of the genotype and enabling the colonization of the new nocturnal niche.^25^^,^^61^

Current artificial lighting at night offers numerous tests of how animals, including geckos, respond to novel light sources.^12^ For instance, six gecko species from the genus *Phelsuma*, although mostly diurnal, shifted their activity from diurnal to nocturnal hours to exploit prey concentrating near artificial lights,^76^ a behavioral change closely resembling that we propose as a potential origin of the Moorish gecko’s nocturnal lineage.

### The influence of human-controlled fire on temporal niche partitioning in Moorish geckos

In conclusion, our investigation into the potential influence of human-controlled fire on the temporal niche partitioning in Moorish geckos presents intriguing insights but also leaves some questions unanswered. The multifaceted evidence we gathered suggests that ancient fires used by humans may have played a role in facilitating the separation between the two gecko variants observed today. However, it does not definitively clarify whether the two lineages originated from diurnal or nocturnal ancestors. The “out of the dark” scenario posits a parsimonious hypothesis where a nocturnal ancestor led to a first nocturnal lineage, followed later by a diurnal descendant, whereas the “into the dark” scenario suggests an alternative origin, where a dark-diurnal ancestor gave rise to a less plastic, more specialized pale-nocturnal lineage. Phenotypic plasticity emerges as a crucial factor in both scenarios, potentially facilitating the colonization of new environments and the subsequent fine-tuning of genetic adaptations.

The ongoing impact of artificial lighting on nocturnal behavior underscores the relevance of our findings, drawing parallels to the adaptation of the Moorish gecko’s nocturnal lineage. Future research could explore further the mechanisms underlying phenotypic plasticity and its interplay with genetic adaptation in this species, shedding light on the evolutionary dynamics of temporal niche partitioning in response to past and current human influences.

### Limitations of the study

Although our work provides evidence supporting the hypothesis of anthropogenic light conditioning in the separation of dark-diurnal and pale-nocturnal gecko populations, it is necessary to clarify which of the two hypotheses (into-the-dark or out-of-the-dark) is the most plausible. Further investigation is needed into the phylogenetics, physiology, and ecology of nocturnal and diurnal forms. Furthermore, while the pattern we described is fascinating and could have potentially occurred in any location with both human communities and geckos in the Mediterranean Basin, it should be verified in additional areas where well-differentiated dark-diurnal and pale-nocturnal populations have been observed. Based on our data, however, we cannot speculate on the existence of such a phenomenon outside the study area.

## Resource availability

### Lead contact

Further information and requests for resources and reagents should be directed to and will be fulfilled by the lead contact, Danilo Russo (danrusso@unina.it).

### Materials availability

This study did not generate new reagents.

### Data and code availability

• The sequencing data are available at National Center for Biotechnology Information (NCBI) as GenBank: MK275668 - MK275678, MK275681, MK275682, MK275684 - MK275686, JQ425045 - JQ425050, JQ425055, JQ425056, JQ425060 and are publicly available as of the date of publication.
• This article does not report original code.
• Any additional information required to reanalyze the data reported in this article is available from the lead contact upon request and are also directly available in the supplementary materials of this article as the distribution of polymorphic gecko populations; phenotypic data on the examined geckos; Homo sapiens past and present habitat suitability map; arthropods captured with and without light.

## Acknowledgments

We thank Greger Larson for his suggestions on a preliminary version of our text. Gaetano Di Pasquale shared important information regarding charcoal analysis and human presence in the study area. We are indebted to two anonymous reviewers for their valuable comments on the first article version.

## Author contributions

Conceptualization: D.F. and M.B.; sample collection: D.F., E.R., V.M., and M.B; laboratory experiments: E.R., V.M., B.A., and M.B; Analyses of data: D.F., D.R., V.M., E.R., B.A., A.M., G.G., and M.B; supervision: D.F.; writing original draft: D.F., E.R., and M.B.; review and editing: D.F., D.R., E.R., V.M., B.A., A.M., G.G., and M.B. All the authors read and approved the article.

## Declaration of interests

The authors declare no conflicts of interest.

## STAR★Methods

### Key resources table

**Table:** 

REAGENT or RESOURCE	SOURCE	IDENTIFIER
**Chemicals**		

MS-222	Sigma-Aldrich	Cat#E10521-10g
Epon 812 resin	Fluka	
Toluidine blue	Sigma-Aldrich	Cat#T3260-5g
Sodium tetraborate buffer solution	Sigma-Aldrich	Cat#B9876-500g
Tris-HCl pH 7.4	Thermo Scientific	Cat#J60202.K2
NaCl	Sigma-Aldrich	Cat#S9888-25G
NP-40	Thermo Scientific	Cat#13434269
Protease inhibitors	Roche	Cat#4693116001

**Deposited data**		

Complete mitochondrial sequence	Fulgione et al., 2019^22^ and this study	National Center for Biotechnology Information (NCBI) accession numbers MK275668 to MK275678, MK275681 - MK275682, MK275684 to MK275686
Partial mitochondrial sequence	Rato et al., 2023^23^	National Center for Biotechnology Information (NCBI) accession numbers JQ425045 to JQ425050, JQ425055, JQ425056, JQ425060

**Software and algorithms**		

Bayesian Evolutionary Analysis Sampling Trees (BEAST)	Bouckaert et al., 2019^77^	
Bayesian Evolutionary Analysis Utility (BEAUti)	Drummond et al., 2012^78^	
Molecular Evolutionary Genetics Analysis version 11 (MEGA11)	Tamura et al., 2021^79^	
Global Biodiversity Information Facility (GBIF) database		www.GBIF.org
R software environment version 4.10	R Core Team 2020	https://www.R-project.org/
iNaturalist		https://www.inaturalist.org/
Tracer v.1.6	Rambaut et al., 2018^80^	
Treeannotator v2.7.7	Drummond et al., 2012^78^	
FigTree v.1.4.4	Rambaut 2010^81^	http://tree.bio.ed.ac.uk/software/figtree/
DensiTree v.2.7.7	Bouckaert et al., 2010^82^	
LogCombiner 2.7.7	Drummond et al., 2012^78^	

**Other**		

Heparinised syringe	N/A	
Yellow sticky traps	N/A	
Super Nova Leica Ultratome	Leica Microsystem	
Leica EZ4 W stereomicroscope	Leica Microsystem	
Zeiss Axioskop 5 microscope	Zeiss	Cat#490980-0001-000
Zeiss Axiocam camera	Zeiss	Cat#426560-9061-000
AvaSpec-2048-USB2-UA-50	Avantes	
Halogen & Deuterium Halogen light sources	Avantes	AvaLight-DH-S
Reflection probe	Avantes	FCR-7UV200-2-ME
White reference tile WS2	Avantes	

### Method details

#### Study area and samples collection

The study was conducted in Southern Italy (40°15′N, 14°54′E, Salerno province) in a region characterised by Mediterranean vegetation, predominantly featuring olive groves, rural structures, and stone walls.

To perform laboratory procedures, we sampled 16 individuals: 6 dark-diurnal geckos collected from tree trunks, and 10 pale-nocturnal geckos captured on walls, using nylon loops. The subjects were then released at the capture site, except for six individuals (three dark-diurnal geckos and three pale-nocturnal geckos) sacrificed to obtain tissues suitable for histological analyses. We sampled Moorish geckos with the approval of the relevant nature conservation authority (Cilento, Vallo di Diano e Alburni National Park, protocol 2013/0010678). The experimental protocols were approved by the Ethical Committee for Animal Experiments of the first author’s University (protocol 2013/0032826).

#### Skin histology

We anesthetized three dark-diurnal and three pale-nocturnal geckos with 250 mg/kg 1% MS-222 (Sigma Chemical Co. St. Louis, MO) injected into the intracoelomic cavity and euthanised them by decapitation. Following this, we processed skin samples taken from the backs of three dark and three light geckos for light microscopy, following the methods outlined in.^83^ In brief, we fixed the samples in 2% paraformaldehyde and 2.5% glutaraldehyde (for 4 h at 4°C) and subsequently post-fixed them in a 2% osmium tetroxide solution (for 1 h at 4°C). After dehydration in an ascending series of ethyl alcohol, the samples were embedded in Epon 812 resin (Fluka). We then cut semi-thin sections of 1.5 μm thickness using a Super Nova Leica Ultratome and stained them with 1% toluidine blue in 1% sodium tetraborate buffer. Finally, we examined the sections using a Zeiss Axiocam camera attached to a Zeiss Axioskop microscope (Zeiss, Jena, Germany).

#### Skin reflectance

To objectively determine the skin coloration of the sampled Moorish geckos, we measured skin reflectance using spectrophotometry (250–1000 nm, AvaSpec-2048-USB2-UA-50; Avantes, Apeldoorn, Netherlands) of the 16 geckos, focusing on the range between 300 and 700 nm.^25^ We employed a white reference tile (WS2; Avantes) as a calibration reference.

The spectrophotometer probe, featuring a 0.2 mm hole end, was positioned perpendicular to the animals' body surface, and the reflectance (R%) was recorded at three locations on their backs. Subsequently, the average of the integrals subtending the reflectance curves was considered representative of the entire back of each individual.^22^^,^^25^^,^^84^ We assessed the difference between the dark-diurnal and pale-nocturnal groups using a one-way ANOVA test, with statistical significance defined as *p* < 0.05.

#### Melanocyte Stimulating Hormone and melanin assay

Plasma and skin samples were obtained from three pale-nocturnal and three dark-diurnal geckos.

Blood was drawn using a heparinised syringe from the interdiscal vertebra windows of the tail. Plasma was then isolated by centrifugation at 2000*g* for 10 min at room temperature, and the samples were stored at −80°C until processing in the laboratory.

We determined the levels of α-MSH using the ELISA assay described by.^71^ Statistical significance differences (defined with *p* < 0.05) between the two groups were assessed using an ANOVA. We followed the protocol proposed by,^85^ with some modifications, to quantify melanin. In brief, tissues were collected in 50 mM Tris-HCl pH 7.4, 300 mM NaCl, 0.5% NP-40, and protease inhibitors (Roche). Cells were lysed using a combination of freeze-thawing (3 cycles of dry ice at −37°C), Dounce homogenisation (200 strokes), and sonication (2 min, 10 s on and 10 s off).

#### Phylogenetic characterisation and time tree

To infer the divergence time of pale-nocturnal and dark-diurnal Moorish gecko populations, we first aligned the mtDNA sequences of *T. mauritanica* from the 6 dark-diurnal (accession number MK275676 to MK275678, MK275684 to MK275686) and the 10 pale-nocturnal specimens (accession number MK275668 to MK275675, MK275681, MK275682) with *T. mauritanica* from Central and Southern Morocco (accession number JQ425056-Marrakech and JQ425055-Anezal),^42^ Iberian Peninsula (accession number JQ425045 - Rambla del Cañar_ Murcia),^42^ and Southern Italy (accession number JQ425046 to JQ425050 and JQ425060),^21^^,^^42^ using “complete deletion” strategies in Molecular Evolutionary Genetics Analysis v.11 (MEGA11).^79^ Then, we constructed a Bayesian phylogeny in Bayesian Evolutionary Analysis Sampling Trees (BEAST) v.1.7.5^77^ with *T. mauritanica* JQ425056-Marrakech as outgroup. We considered General Time Reversible (GTR) substitution model as suggested by “find best DNA model” option in MEGA11.^79^ We used a strict clock model with the default parameters and default operators set in Bayesian Evolutionary Analysis Utility (BEAUti)^78^ to estimate the divergence times.

Because no suitable fossil record exists for calibrating the mutation rate in *T. mauritanica*, we adopted a substitution rate of the mtDNA of lizards (5.29 × 10^−9^).^86^ Each Markov chain Monte Carlo (MCMC) sample was based on a run of 100,000,000 generations and sampled every 1,000 generations. The first 10% of samples were treated as burn-in and removed before individual runs were combined in LogCombiner v.2.7.7.^78^ Stationarity was assessed using the program Tracer v.1.6^80^ with ESS values >200 taken as evidence for convergence. A final MCMC maximum clade credibility tree was generated from the cumulative post-burn-in sample of the combined analyses in the program Treeannotator v2.7.7.^78^ The resulting consensus tree was visualized using FigTree v.1.4.4^81^ and the distribution of the trees was visualized with DensiTree v.2.7.7,^82^ using the same parameters described above, with burn-in of 10%.

#### Potential human-gecko co-occurrence

To establish whether Moorish geckos and humans coexisted when the dark-diurnal and pale-nocturnal geckos split, we generated Species Distribution Models (SDM) for *T. mauritanica* and projected them to the past. We collected modern occurrence data for the gecko from the Global Biodiversity Information Facility (GBIF) database by selecting “human observation” and “machine observation” as the basis of the record (www.GBIF.org, 26 January 2024; GBIF Occurrence Download https://doi.org/10.15468/dl.xprybx). The data were further filtered by excluding occurrences without geographical coordinates. For the human dataset, we used the *H. sapiens* records published by^87^ and,^88^ focusing on fossil presence observations dated within a time window coherent with the divergence time estimates between the gecko lineages we found (see below). Radiocarbon data were calibrated using the “Bchron R” package^89^ through the “intcal20” curve^90^ in R environment.^91^ As environmental predictors, we employed the monthly bioclimatic variables generated through the 2Ma CESM1.2 simulation,^88^ downscaled at a 0.5 ° × 0.5 ° grid resolution. The native set of predictors was converted into bioclimatic variables according to WorldClim using the “dismo” R package.^92^ Lastly, variables were projected onto the Mollweide coordinate reference system. To prevent model overfitting, duplicate occurrences in the raster grid were removed. After this step, we gathered 520 modern *T. mauritanica* and 740 past *H. sapiens* occurrences.

We defined the Mediterranean region as the study area for both *H. sapiens* and *T. mauritanica*, according to the current and historical geographical distribution of the Moorish gecko.^21^ Then, we randomly generated 10,000 background points within the study area. To account for potential sampling biases, pseudoabsences were geographically placed according to the density of the occurrence data, making them more abundant where presences are denser.^93^^,^^94^^,^^95^ For *H. sapiens* only, the record was divided into 1000-year consecutive time bins according to the time resolution of bioclimatic predictors. Subsequently, we partitioned the pseudoabsences proportionally to the number of presences per time bin. After extracting climatic values at each occurrence and pseudoabsence data point, we accounted for multicollinearity among predictors by considering the Variance Inflation Factor (VIF). The latter was assessed using the function “vifcor” embedded in the “usdm” R package, selecting a threshold of 0.75.^96^^,^^97^ After applying VIF, the selected predictors were BIO4 (temperature seasonality), BIO8 (mean temperature of the wettest quarter), BIO9 (mean temperature of the driest quarter), BIO13 (Precipitation of Wettest Month), BIO14 (precipitation of the driest month), BIO15 (Precipitation Seasonality), and BIO19 (precipitation of the coldest quarter).

To estimate the geographic distribution of both species in the past, we adopted the SDM ensemble approach. Specifically, we trained SDMs through an ensemble forecasting approach, using the R package “biomod2”.^98^ We considered four different algorithms: Maximum Entropy Models (MaxEnt), Generalized Boosted Models (GBM), Random Forests (RF), and Generalized Linear Models (GLM). We adopted the default settings described in the “biomod2” R package for model tuning. To evaluate the predictive accuracy of SDMs, we randomly split the dataset into a 70% sample used for model calibration and the remaining 30% used to assess model performance. Then, we calculated the area under the receiver operating characteristic curve (AUC;^99^) and the true skill statistic (TSS;^100^). This procedure was iterated 10 times, changing the randomly selected training/testing data points at each iteration. Model averaging was performed by weighting the individual model projections by their AUC values and averaging the results,^101^ excluding the model with AUC <0.7. Lastly, SDM predictions were projected into the past to obtain the potential distribution of *T. mauritanica* in the Mediterranean area during the time interval predicted by the phylogenetic reconstruction as the divergence time between the two Moorish gecko forms.

To test whether there was a significant spatial correlation between *H. sapiens* and *T. mauritanica*, we adopted the Boyce Index.^98^ This index requires only presence data points and measures how much model predictions differ from a random distribution of observed presences across prediction gradients.^99^ The Boyce Index ranges from −1 to +1, with positive values indicating a model whose predictions are consistent with the distribution of presences in the evaluation dataset. Values close to zero mean that the model does not differ from a random model, and negative values indicate counter predictions, i.e., predicting poor-quality areas where presences are more frequent.^98^

We used the Boyce Index to test whether the predicted suitability for *T. mauritanica* is higher in the presence of humans than otherwise throughout the study area. In addition to the Boyce Index, we trained a Linear Mixed-Effects Model to test whether the suitability of the two species is associated, setting the variable “time” as a random effect. This was done using the “lme4” R package.^102^ To provide a visual rendering of the potential spatial overlap between the two species across space and time, we first binarised the individual species prediction maps by adopting the threshold that maximises the sum of sensitivity and specificity (“MaxSens + Spec”). We then stacked the binary maps of the two species at each 1 kyr according to the time resolution of the bioclimatic variables. Map binarisation was performed using the “PresenceAbsence” R package.^103^

#### Testing differences in prey availability due to fire

We conducted a paired-design experiment to assess the power of fire in attracting arthropods at night, affecting food availability for Moorish geckos. We selected four light-coloured walls, resembling rock surfaces, and compared the number of arthropods lying on each wall over 5 hours when fire was set nearby *vs* a “dark” (no fire) control. The distance of the fire from the wall was carefully calibrated to prevent excessive heating while ensuring a broad surface of light. The flame never exceeded 40 cm in height, was 30 cm wide and was fuelled by dry wood collected from the surrounding area. The night of treatment was randomly assigned.

To collect arthropods comprehensively, we hung to each wall fifteen 15x20cm yellow sticky traps regularly spaced over the wall surface. Besides, we also collected arthropods lying in the spaces between sticky traps using hand nets and tweezers. We identified all collected arthropods using a Leica EZ4 W stereomicroscope (Leica Microsystems) following^104^^,^^105^ and using local reference collections. Identification was done at the order level and all individuals were counted. We used a paired-sample t-test to compare the number of individuals captured by order between lit *vs* unlit walls. The results was considered statistically significant with p<0.05.

### Quantification and statistical analysis

To test differences in skin reflectance, dermal melanin content and α-MSH plasmatic levels between pale-nocturnal and dark-diurnal geckos, we performed an one-way ANOVA test in R (version 4.3.0) considering p<0.05 as statistically significant. The results of significance are reported in the main text (Result section) and legend of Figure 2.

To evaluate the spatial correlation between humans and the Moorish gecko in the past, we used the Boyce Index, the Spearman correlation test and the linear mixed-effects model, with p<0.001 considered statistically significant. The results are reported in Figure 4.

To test the differences in arthropods sampled at unlit *versus* fire-lit walls, we conducted a paired two-tailed t-test, with p<0.05 considered statistically significant. The results are reported in the legend of Figure 6 and in Table 1.

## Supplemental information

Document S1. Figures S1 and S2 and Tables S1–S3

Table S4. Excel file linked with Figure 4 shows evidence of human presence at the epoch of the split observed in Tarentola mauritanica AMS, Accelerator mass spectrometry; 14C, Radioncarbon dating; OSL, Optical stimulated luminescence.

Table S5. Arthropods (number of individuals) captured on unlit walls (NF) and illuminated walls (F) sorted by Order, related to Figure 6
