# Supplementary material for: Flame-forged divergence? Ancient human fires and the evolution of diurnal and nocturnal lineages in moorish geckos
Source: iScience. 2024 Dec 30;28(2):111715. doi: 10.1016/j.isci.2024.111715 (PMC11783447; doi:10.1016/j.isci.2024.111715)
Supplement: Document S1. Figures S1 and S2 and Tables S1–S3 [file mmc1.pdf]

iScience, Volume *28*

Supplemental information

Flame-forged divergence? Ancient human fires and the evolution of diurnal and nocturnal lineages in moorish geckos

Domenico Fulgione, Danilo Russo, Eleonora Rivieccio, Valeria Maselli, Bice Avallone, Alessandro Mondanaro, Giorgio Giurato, and Maria Buglione

Supplemental Information

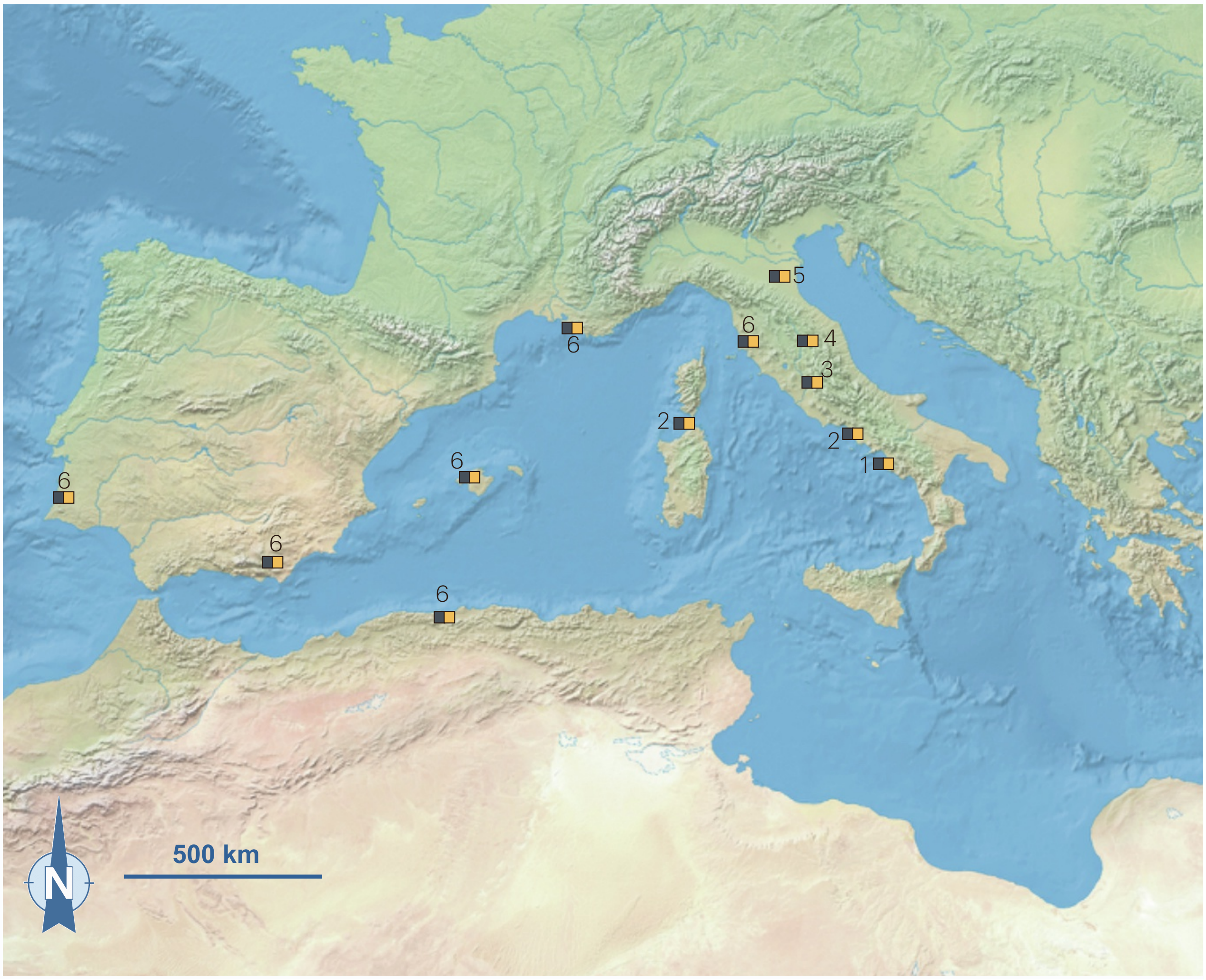

Figure S1. Populations of Moorish gecko showing the two morphotypes (dark-diurnal and pale-nocturnal) recorded across the Mediterranean Basin (related to Figure 1).

1) Study area.

2) Other observations by the authors.

3) Simbula et al. (2018).

4) Cristiano Spilinga (pers. Comm.) Tuoro sul Trasimeno (Umbria, Central Italy).

5) Venice (from photograph by Nicolò Bertaglia).

6) iNaturalist records (https://www.inaturalist.org/). See https://www.inaturalist.org/observations?taxon_id=33602 for more examples.

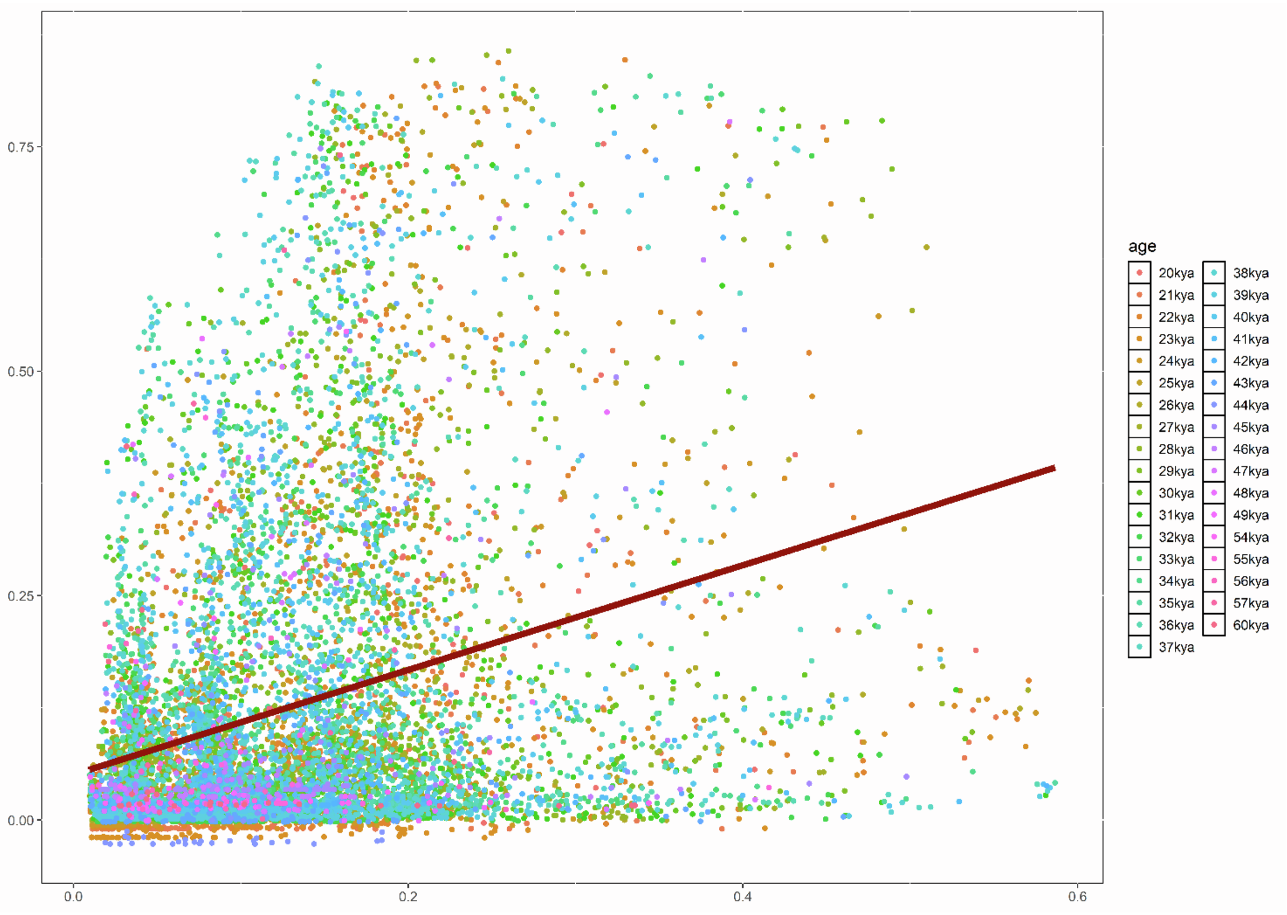

Figure S2. Relationship between human *(Homo sapiens*) and Moorish gecko (*Tarentola mauritanica*) potential distribution values fitted by the Linear Mixed-Effects Model. The dark red line represents the regression line obtained by collapsing all the conditional fits from the random effect model into a single marginal fit (fixed effect). Angular coefficient of the distribution, a = 0.6. Coloured points represent the marginal model fits plus the conditional residuals (related to Figure 4).

Table S1. Reflectance values betweeen 200-700 nm for each point of measurement for pale-nocturnal (N1-N10) and dark-diurnal (D1-D6) geckos. Related to Figure 2. SD, standard deviation.

	**reflectance value (200-700nm)**							
**Sample**	**Point_1**	**Point_2**	**Point_3**	**Point_4**	**mean reflectance values (200-700nm) single gecko**	**SD**	**mean reflectance values (200-700nm) pale-nocturnal gecko population**	**SD**
**N1**	2912.24	2980.61	4652.16	2293.73	3209.685	874.7366	2317.26825	523.496
**N2**	2282.05	1787.83	2623.72	4842.42	2884.005	1169.09		
**N3**	2719.11	2378.58	2914.92	2513.61	2631.555	203.6351		
**N4**	2098.92	2028.48	3568.47	2646.71	2585.645	615.8344		
**N5**	2714.74	2104.14	2782.42	2241.96	2460.815	292.8405		
**N6**	2144.82	2013.29	2011.99	2292.69	2115.6975	115.5606		
**N7**	2827.57	1308.51	2014.01	1769.84	1979.9825	551.0439		
**N8**	2229.81	2047.93	1924.46	1640.64	1960.71	214.3484		
**N9**	2169	2143.16	1584.34	1385.39	1820.4725	343.0211		
**N10**	1594.32	1348.85	1712.96	1440.33	1524.115	139.9365		
							**mean reflectance values (200-700nm) dark-diurnal gecko population**	**SD**
**D1**	930.11	358.61	821.3	1062.35	793.0925	264.9736	566.3316667	61.4517
**D2**	656.73	703.17	804.48	483.32	661.925	116.1355		
**D3**	672.54	497.5	398.03	945.07	628.285	207.6255		
**D4**	240	600	575.38	400	453.845	145.5687		
**D5**	664.06	326.92	218.64	512.91	430.6325	170.9935		
**D6**	431.71	79.37	819.35	390.41	430.21	262.7284		

Table S2. Amount of melanin in the total protein extract of the skin of Moorish geckos (*Tarentola mauritanica*). D, dark-diurnal geckos; P, pale-nocturnal gecko. Related to Figure 2. SD, standard deviation.

	**Abs 492nm/ug protein**	**Abs 492nm/ug protein**	**Abs 492nm/ug protein**	**mg protein**	**mean Abs 492nm/ug protein**	**SD**	**Mean**	**SD**
**D1**	11.40	10.50	7.90	11	9.93	1.82	8.189	1.543565
**D2**	8.10	8.10	6.70	16	7.63	0.81		
**D3**	6.50	6.40	8.10	11	7.00	0.95		
**P1**	5.01	5.19	4.57	14	4.92	0.32	4.81	0.125352
**P2**	5.46	4.48	4.52	20	4.82	0.55		
**P3**	4.98	4.95	4.10	15	4.68	0.50		

Table S3: α-MSH concentration (mg/ml) in pale - nocturnal (NOC 1-3) and dark-diurnal (DIU 1-3) Moorish geckos (*Tarentola mauritanica*). Related to Figure 2. SD, standard deviation.

**samples**	**Abs 450nm**		**mean**	**delta**	**ug prot tot**	**ul/well**	**mg/ml**	**ng/mg prot tot**	**Mean**	**SD**
ng/well	rep1	rep2								
B	0.083	0.087	0.086							
**NOC1**	0.287	0.283	0.285	0.199	200	7.27	0.55	19929.08	0.42	0.15
**NOC2**	0.154	0.148	0.151	0.065	200	5.00	0.26	6508.70		
**NOC3**	0.224	0.231	0.228	0.142	200	6.36	0.44	14150.00		
**DIU1**	0.357	0.388	0.373	0.287	200	5.45	1.05	28653.51	1.38	0.29
**DIU2**	0.628	0.712	0.670	0.584	200	7.27	1.61	58425.23		
**DIU3**	0.485	0.500	0.492	0.406	200	5.45	1.49	40636.90		
